# Ifosfamide-loaded poly (lactic-co-glycolic acid) PLGA-dextran polymeric nanoparticles to improve the antitumor efficacy in Osteosarcoma

**DOI:** 10.1186/s12885-015-1735-6

**Published:** 2015-10-21

**Authors:** Bin Chen, Jie-Zuan Yang, Li-Feng Wang, Yi-Jun Zhang, Xiang-Jin Lin

**Affiliations:** 1 Department of Orthopedic, The First Affiliated Hospital of Medical School of Zhejiang University, No. 79 Qingchun Road, Hangzhou, Zhejiang 310003 China; 2 Department of Laboratoire Central, The First Affiliated Hospital of Medical School of Zhejiang University, Hangzhou, 310003 China

**Keywords:** Ifosfamide, Osteosarcoma, Polymeric nanoparticles, Block copolymer, Apoptosis

## Abstract

**Background:**

Osteosarcoma is a typical bone cancer that primarily affects adolescents. The therapeutic activity of drugs is limited by their severe drug-related toxicities, therefore, a therapeutic approach which is less toxic and highly effective in tumor is of utmost importance.

**Method:**

In this study, ifosfamide-loaded poly (lactic-co-glycolic acid) (PLGA)-dextran polymeric nanoparticles (PD/IFS) was developed and studied its anticancer efficacy against multiple osteosarcoma cancer cells. The drug-loaded nanoparticle was characterized for physical and biological characterizations.

**Results:**

The formulated PD/IFS showed a high drug loading capacity and displayed a pH-sensitive release pattern, with a sustained release profile of the IFS. PD/IFS nanoparticles exhibited remarkable in vitro anticancer activity comparable to that of free IFS solution in a concentration dependent manner in MG63 and Saos-2 cancer cells. PLGA-dextran by itself did not affect cell viability of cancer cells indicating its excellent biocompatibility. The formulation exhibited significantly higher PARP and caspase-3/7 expression in both the cancer cells.

**Conclusion:**

Our study successfully demonstrated that nanoparticulate encapsulation of antitumor agent will increase the therapeutic efficacy and exhibit a greater induction of apoptosis and cell death.

## Background

Osteosarcoma (OS) is one of the typical bone cancers that occur in distal femur and proximal tibia [1]. OS being mesenchymal in nature are very aggressive and more than 20 % of cases are diagnosed at metastatic stage. Specifically, OS is commonly seen in children and adolescents [2]. Parallel to other solid tumors, OS tumors also contains a highly heterogeneous population of cancer cells in terms of growth rate, karyotype, antigenicity and chemosensitivity. Although 5-year survival rate increased to 65 %, yet it is way behind the overall cancer survival rate [3, 4]. Furthermore, survival rate of 5-year metastatic disease is still at a meager 20 %. At present, the therapies for OS treatment include surgical resection followed by chemotherapy regimens of various drugs including doxorubicin, cisplatin, and ifosfimide [5]. However, therapeutic activity of these drugs is limited by their severe drug-related toxicities such as cardiotoxicity and nephrotoxicity. Therefore, a therapeutic approach which is less toxic and highly effective in tumor is of utmost importance [6]. In this regard, present research is mainly focused on developing unique and novel therapeutic carriers to deliver the chemotherapeutic drugs to the cancer cells.

Ifosfamide (IFS) is a DNA-alkylating agent and a structural analog of cyclophosphamide. It acts as a prodrug, its metabolism occurring mainly through CYP 3A4 and CYP 2B6 enzymes, which are present predominantly in the hepatocytes [7, 8]. IFS crosslinks DNA strands and inhibits DNA replication and ultimately leads to apoptosis due to activation of caspases in the cells. IFS is indicated as a mainline treatment for OS and delivered as an intravenous infusion [9]. A variety of nanoparticle-based delivery systems have been developed for the delivery of anticancer drugs. Self-assembled polymeric nanoparticles, have received increased attention for their potential application in biotechnology and medicine, especially as a drug delivery carrier in cancer therapeutics [10]. These amphiphilic nanoparticles usually have a hydrophobic core shielded by a hydrophilic shell when present in the aqueous environment. The hydrophobic core involves in the drug incorporation and the outer hydrophilic shell prevents the delivery system against reticuloendothelial system (RES) [11]. The polymeric self-assembled nanoparticles offer some unique advantages including core-shell morphology, high loading capacity, site-specific drug delivery, and avoids unwanted side effects of administered drug. Moreover, micelles remain stable in blood circulation for prolonged period of time and could avail enhanced permeability and retention effect (EPR) based passive targeting [12, 13].

Dextran, a polysaccharide is characterized as a colloidal and hydrophilic substance [14]. Dextran is extensively employed as a delivery carrier owing to its excellent biocompatible and immunoneutral properties. Moreover, hydroxyl group present in the glucose unit allow for easy chemical conjugations [15]. Biodegradable polymer, poly(lactic-co-glycolic acid) (PLGA) was selected due to its excellent systemic characteristics and biodegradability. Several studies have reported that nanosized PLGA NP would be in the ideal range of EPR effect as well as to avoid reticuloendothelial system (RES) mediated clearance. However, delivery characteristics of PLGA could be further improved by conjugating with hydrophilic dextran sulphate (DS) [16]. Recently, Jeong et al. reported that PLGA-dextran block copolymer forms self-assembling nanoparticles and could be used as a carrier to deliver multiple anticancer agents [17]. Consistently, we have synthesized a PLGA-dextran block copolymer via EDC/NHS chemistry and encapsulated IFS. We expected that incorporation of IFS in PLGA-dextran based polymeric nanoparticles will effectively increase the chemotherapeutic efficacy in cancers while at the same time reduce the overall side effects.

Thus far, the main aim of this study was to prepare ifosfamide-loaded PLGA-dextran polymeric nanoparticles for the treatment of osteosarcoma (OS). We hypothesized that IFS incorporation in a nanocarrier would increase its therapeutic effect due to the controlled release and defined properties. The dynamic light scattering analysis and morphology analysis were carried out to optimize the formulations. The biocompatible nature of blank nanoparticles (NP) and cytotoxic effect of IFS-loaded NP was evaluated in MG63 and Saos-2 osteosarcoma cells via MTT assay. The apoptotic effect of free drug and IFS-loaded NP was studied means of PARP and caspase-3, which are typical apoptotic markers.

## Materials and methods

### Materials

Ifosfamide (≥98 %) was purchased from Sigma Aldrich (St. Louis, MO, USA).Poly(d,l-lactic-co-glycolic acid) (PLGA) (Mw: 10,000; lactic acid : glycolic acid = 50:50) was procured from Wako Pure Chemical (Tokyo, Japan). Dextran from Leuconostocspp was also obtained from Sigma-Aldrich (China). All other chemicals were reagent grade and used without further purifications.

### Synthesis of PLGA-Dextran block copolymer

Approximately 3 g of PLGA-COOH was dissolved in anhydrous methylene chloride and to this organic solution, 70 mg of NHS (N-hydroxysuccinimide) and 140 mg of EDC (1-ethyl-3-(3-dimethylaminopropyl)-carbodiimide) was added. The organic mixture was stirred continuously for 12 h at room temperature. The 12 h time period is sufficient for the complete activation of carboxylic acid group in PLGA. The formed PLGA-NHS was precipitated by the addition of ice cold ether, washed with organic solvent mixture, and dried.

Aminated dextran was prepared as reported previously. Briefly, dextran and cyanoborohydride was mixed in a DMSO medium and to this mixture hexamethylene diamine was added and allowed the reaction for 24 h. The amine group terminated dextran was collected, dialyzed, and lyophilized. To prepare the block copolymer, 100 mg of PLGA-NHS and 125 mg of dextran was dissolved in DMSO and inert atmosphere was maintained throughout the reaction time. The formed PLGA-dextran was dialyzed using dialysis membrane (molecular weight cutoff, 10,000 g/mol) for 3 days. The resulting products was lyophilized and dried under vacuum conditions.

### Preparation of Ifosfamide-loaded polymeric nanoparticles

IFS-loaded polymeric nanoparticles (NP) were prepared by precipitation method. In brief, 25 mg of PLGA-dextran (PLD) and 5 mg of IFS were dissolved in 5 ml of DMSO and to this mixture 20 ml of ultra-pure water were added. The mixture was magnetic stirred for 2 h and followed by dialysis against distilled water. The dialysis process was continued for 3–4 h and the resulting drug-loaded polymeric NP was collected and lyophilized.

### Drug loading

The loading efficiency and loading capacity was determined as follows. In brief, 10 mg of lyophilized NP was dissolved in 5 ml of DMSO and sonicated for 15 min. The organic solution was centrifuged and the supernatant was used to calculate the amount of drug loaded. The drug loading was quantified using HPLC method. The HPLC system (Shimadzu, Kyoto, Japan) consisted of LC-10AT pump, a SPD-10A UV/Vis detector and a DGU-14A degasser model. The flow rate was maintained at 1 ml/min. The wavelength of detection was 254 nm. 50 mM of KH _2_ PO _4_ (pH 5.0) was used as a mobile phase.

### Particle size and size distribution analysis

The average particle size and size distribution analysis was performed using a Zetasizer Nano-S90 (Malvern Instruments, Malvern, UK) and a 633 nm He-Ne laser beam at a fixed scattering angle of 90°. A dilute solution of NP was used to analyse the particle size. The experiments were performed in triplicates.

### Transmission electron microscopy

The morphology of the PD/IFS was examined on a transmission electron microscope (JEOL JEM-200CX). Before the examinations, NP dispersion was diluted many times with ultra-pure water. The aqueous solution was dropped on the carbon coated copper grid and counter stained with 2 % phosphotungistic acid. The samples were dried using an infrared lamp and viewed under TEM.

### Drug release study

The IFS release from the PD/IFS NP system was determined using a dialysis method. Briefly, 30 mg of PD/IFS lyophilized powder was dissolved in 1 ml of water and sealed in a dialysis tube. The dialysis tube was in turn placed in a 50 ml of Falcon tube containing 25 ml of release media. Selective release media including phosphate buffered saline (PBS, pH 7.4) and acetate buffered saline (ABS, pH 5.5) was used. The main reason behind the selection of different pH was to mimic the conditions of tumor microenvironment. The sampling was done at specific time points such as 1,2,4.6,8,10,12,24,48,72,96,120 h. At each sampling point, 1 ml of release sample was withdrawn and replaced with equal volume of fresh media. The released IFS content in the released medium was determined by HPLC as previously described.

### Cell culture

MG63 and Saos-2 osteosarcoma cancer cells were grown in DMEM supplemented with 10 % FBS, 100 units/mL penicillin and 100 μg/mL of streptomycin. Cells were maintained at 37 °C with 5 % CO2 in a humidified incubator.

### Cell viability assay

Cell viability was assessed using 3-(4,5-Dimethylthiazol-2-yl)-2,5-diphenyltetrazolium bromide (MTT) calorimetric assay. MG63 and Saos-2 osteosarcoma cancer cells were seeded in a 96-well plate (4000 cells/well) and allowed to grow for 48–72 h. Next day, media was removed and replaced with fresh media containing blank PLGA-dextran, free IFS, and PD/IFS NP in a concentration-dependent manner. The formulations were incubated for 24 h and cell viability was estimated using MTT solution. MTT reagent 20 μL in PBS was added into each well and the plate was incubated for 4 h at 37 °C. The culture medium in the wells was removed and 200 μL of dimethylsulfoxide (DMSO) was added into the wells. The optical density of the solution was measured at 570 nm with a microplate reader. The mean value and standard deviation for each treatment were determined and then converted values relative to the control. IC50 were calculated using GraphPad Prism software.

### Morphological cell imaging

Cover slips were rinsed in 70 % ethanol for 10 min and washed with PBS. The cells were seeded into the cover slips and allowed to attach for 12 h. The formulations as mentioned above was added to each well and further incubated for 24 h. Then samples were washed with PBS, fixed with formalin (Sigma), and viewed under Nikon Eclipse 60i microscope system.

### Caspase-3 activity

The activity of caspase-3 was measured by colorimetric assay kits (Sigma-Aldrich) as per the manufacturer’s protocols. MG63 and Saos-2 osteosarcoma cancer cells were seeded in a 6-well plate (1 × 10^6^ cells/well) and allowed to attach for 24 h. Next day, media was removed and replaced with fresh media containing blank PLGA-dextran, free IFS, and PD/IFS NP in a concentration-dependent manner. The cells were incubated with respective formulations for 24 h. Cell pellets were collected and treated with lysis buffer and incubated for 10 min in ice bath. The lysate was collected, centrifuged and supernatant was collected and evaluated for caspase-3 activity.

### Apoptosis analysis

FACS analysis is considered to be a specific and objective method for quantitative determination of apoptosis. MG-63 and Saos-2 cells were seeded at a density of 5 × 10^5^ cells in a 6-well plate and incubated for 24 h. When the cells reached 80 % confluence, cells were treated with free IFS, and PD/IFS NP formulations (1 μg/ml) and further incubated for 24 h. Following day, cells were harvested, washed, and incubated with a mixture of 0.25 mg/mL Annexin-V FITC and 10 mg/mL PI. The mixture was kept for 15 min at 37 °C. Excess PI and AV-FITC fluorescence were then washed off and cells were measured by flow cytometry (FACS Calibur, BD Biosciences). A minimum of 10,000 events was counted per sample by flow cytometry.

### Statistical analysis

Results in the present study are presented as means ± standard deviations. Statistical significance was evaluated by analysis of variance (ANOVA), followed by Tukey’s post-hoc test. *P-values of *p* < 0.05 was considered to be statistically significant.

## Results

### Characterization of PD/IFS nanoparticles

PLGA-dextran formed self-assembled polymeric micelles in the aqueous medium. Generally, PLGA is hydrophobic, so it should form the inner core of the polymeric micelle while the dextran domain should form the outer shell due to its hydrophilic nature [18]. Polymeric micelles incorporated drugs by the hydrophobic interaction between the drug and the hydrophobic domain of the block copolymer. It has been frequently reported that polymeric micelles enhances accumulation in tumor cells and prolongs blood circulation times [19].

### Particle size analysis

**Fig. 1:**
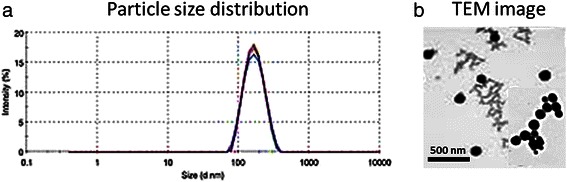
**a** Particle size distribution of ifosfamide-loaded PLGA-dextran (PD/IFS) nanoparticles **b** TEM image of PD/IFS

The particle size and size distribution of PD/IFS NP was investigated by means of dynamic light scattering (DLS) technique. The particle size of PD/IFS was observed to be 124 ± 3.45 nm with an excellent dispersity index of 0.124 (PDI) (Fig. 1a). Blank polymeric micelles posted an average size of 75 ± 2.39 nm. The increase in particle size upon drug incorporation might due to the bulkier core of micellar system. Furthermore, it has been frequently reported that small particle size <200 nm could accumulate preferentially in the tumor tissues via enhanced permeability and retention (EPR) effect. Other than this, small particle size could effectively evade the RES based clearance system in the blood circulation [19].

### Morphological analysis

The particle size of PD/IFS NP was further confirmed by TEM imaging. As seen from Fig. 1b, particle sizes were in the range of 60–80 nm and uniformly distributed in the carbon coated copper grid. The particles were clearly spherical and present as a dense black object the TEM grid. No apparent sign of aggregation was seen among the particles. It has to be noted that particle size observed form TEM was smaller than observed from DLS analysis. The difference in particle size might be attributed to the dried state (from TEM) and hydrated state (from DLS) of particles.

### Drug loading and In vitro drug release

**Fig. 2:**
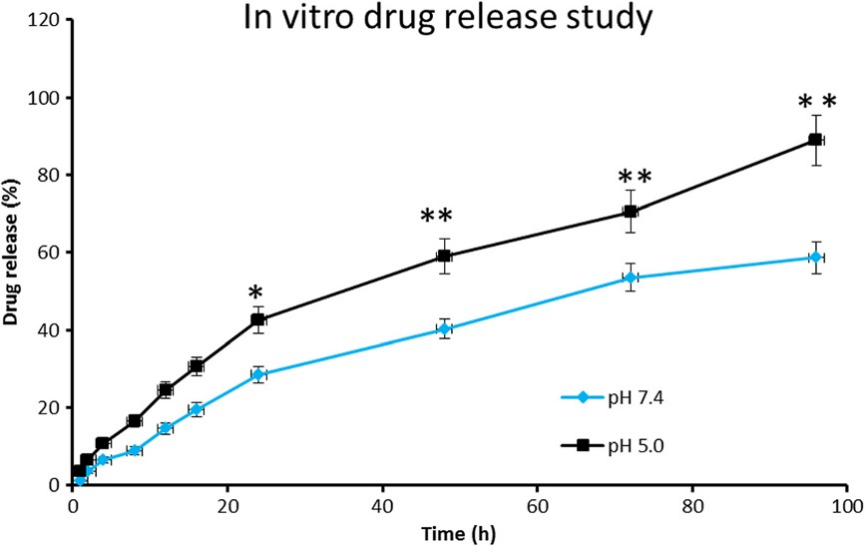
The release profile of IFS from PLGA-dextran nanoparticulate system. The release study was performed in phosphate buffered saline and acetate buffered saline. The study was carried out for 96 h.**p < 0.01 is the statistical difference between pH 7.4 and pH 5.5 release medium

IFS was effectively entrapped in the NPs with a loading and encapsulation efficiency of 20.15 ± 3.5 % and 89 ± 1.95 %, respectively. The release profile of IFS from PD/IFS NP was performed in phosphate buffered saline (PBS) and acetate buffered Saline (ABS) at 37 °C. Results showed that IFS released in a sustained manner throughout the study period up to 96 h (Fig. 2). As expected, PD/IFS showed a pH-dependent release profile with accelerated release in the acidic pH than comparing to that of physiological pH conditions. It has to be noted that accelerated release of drug from the NP might be attributed to the fast diffusion of drug and partially due to the higher degradation of delivery vehicle in the acidic conditions. Broadly, release profile of IFS in pH 7.4 and pH 5.0 could be divided into two parts; first, faster release of IFS was observed until 24 h and second, a relatively more sustained release phenomenon was observed from 24 to 96 h study period. For example, nearly ~30 % of IFS released in first 24 h while only ~55 % of drug released by the end of 96 h in PBS media. Similar trend was observed in ABS media, where nearly ~40 % of drug released in24h and completed the release (100 %) by the end of 96 h. The sustained release of drug in pH 7.4 condition and accelerated release in pH 5.0 conditions would be advantageous in cancer drug delivery.

### Cytotoxicity assay and cellular morphology

**Fig. 3:**
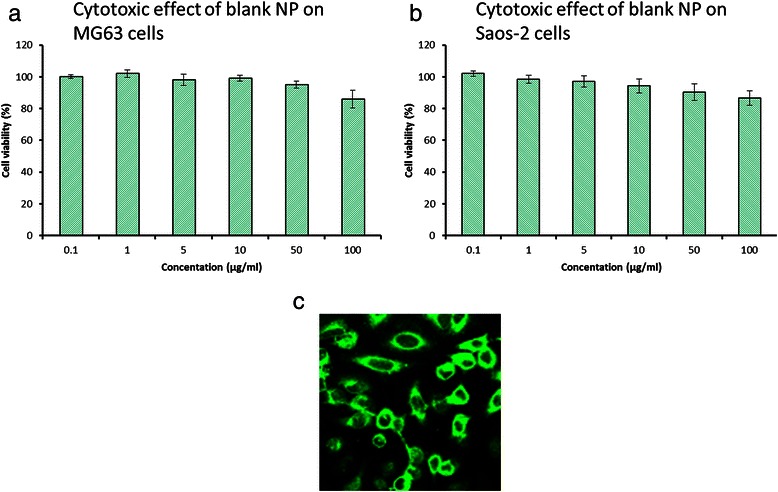
Cytotoxicity assay for blank nanoparticles in **a** MG63 **b** Saos-2 osteosarcoma cancer cells. **c** Confocal laser scanning microscopic images of PD/IFS

The cancer cells were treated with blank NP with different concentrations ranging from 0.1 to 100 μg/mL (Fig. 3a, b). The results clearly showed that synthesized polymers were highly biocompatible and showed a cell viability of more than 90 % throughout all the concentrations tested. Fluorescent images of MG63 cells showed that cells maintained their morphology when incubated with blank NP (Fig. 3c). The polymeric carrier itself did not contribute to cytotoxicity is very advantageous.

**Fig. 4:**
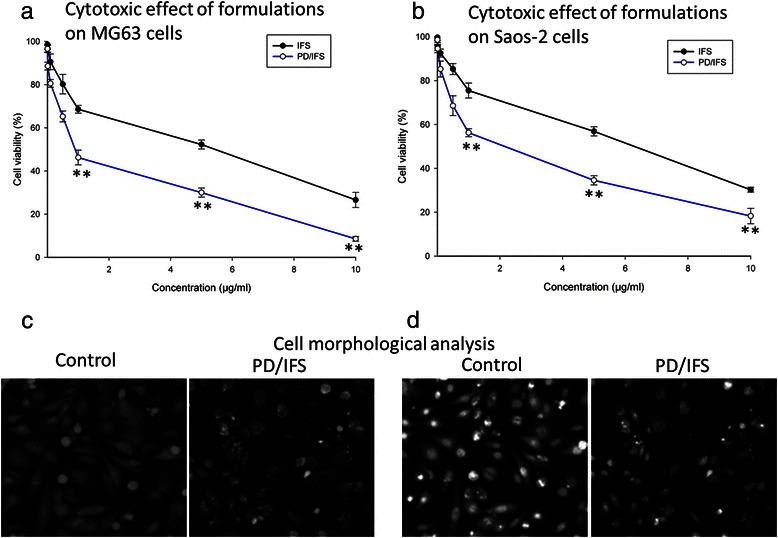
Cytotoxicity of free IFS and PD/IFS (with equivalent IFS concentration) on (**a**) MG63 (**b**) Saos-2 osteosarcoma cancer cells. The cytotoxicity assay was performed by MTT technique. The cells were incubated for 24 h and the experiment was repeated four times in triplicate. Optical images of (**c**) MG63 (**d**) Saos-2 cells after incubation with free IFS and PD/IFS for 24 h. **p < 0.01 is the statistical difference between IFS and PD/IFS in both cancer cells

Cytotoxic potential of free IFS and PD/IFS was evaluated in both the osteosarcoma cancer cell lines. The cells were cultured in the presence of free IFS and PD/IFS NP at increasing concentrations of drugs. As shown in Fig. 4a, b, both free IFS and PD/IFS were able to effectively inhibit cell growth and showed a concentration dependent-cytotoxic effect.

Furthermore, morphology of cells treated with free IFS and PD/IFS NP was evaluated by optical microscope. In both the case, untreated cells presented a well-defined morphology and adhered to the cover slip in the 6-well plate (Fig. 4c, d). In case of PD/IFS treated group, marked presence of dead cells were observed. The cells were either fusiform or rounded and in the process of dying indicating the cytotoxic effect of the optimized formulations.

### Cellular apoptosis analysis

**Fig. 5:**
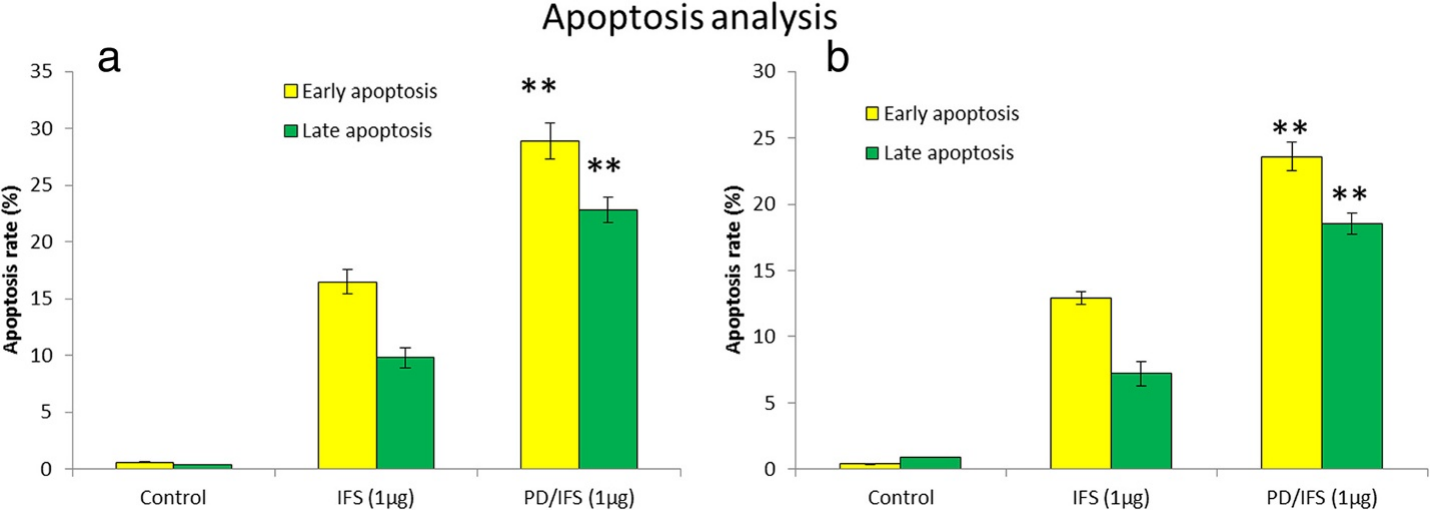
Apoptosis analysis was detected by Annexin-V/PI staining. Apoptosis of **a** MG63 **b** Saos-2 cancer cells. The respective cell percentages in early and late apoptosis for different time period are presented in the bar graph. **p < 0.01 is the statistical difference in apoptosis between IFS and PD/IFS in both cancer cells

**Fig. 6:**
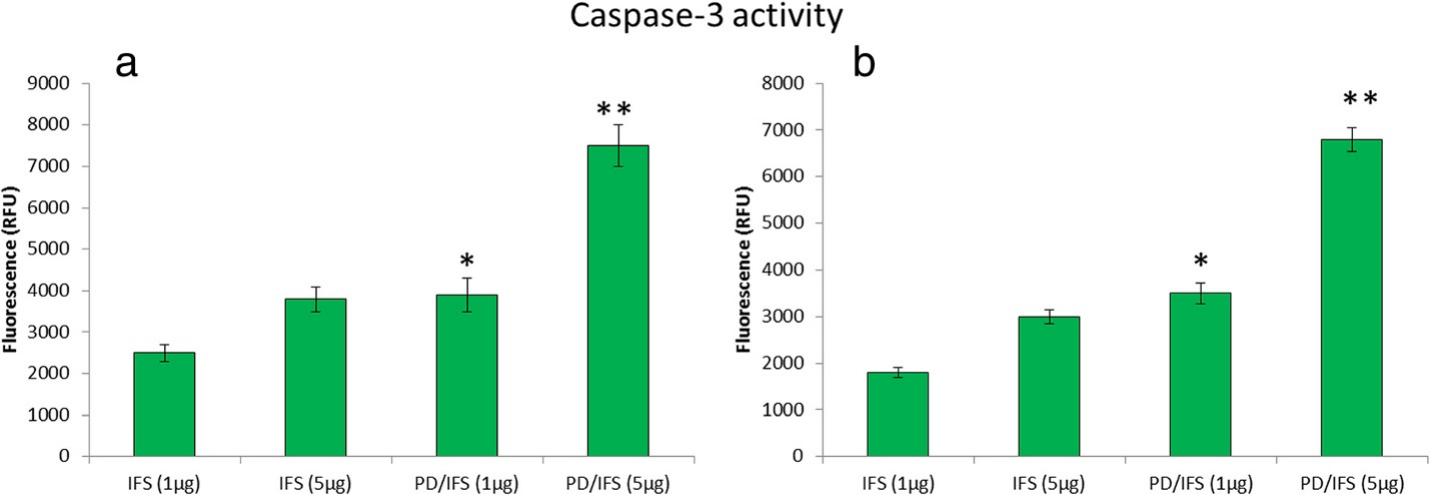
Caspase-3activity was measured as a second parameter of apoptotic cell death in **a** MG63 **b** Saos-2 cancer cells. Significant increase in apoptosis was observed when they were treated with IFS loaded nanoparticle. *p < 0.05 and **p < 0.01 is the statistical difference between IFS and PD/IFS in both cancer cells

The ability free IFS and PD/IFS NP to induce apoptosis on representative MG63 and Saos-2 cancer cell was evaluated by means of Annexin-V/PI-mediated apoptosis analysis. It can be clearly seen (Fig. 5a, b) that PD/IFS induced a greater apoptosis rate in both the cancer cells. Caspase-3 activity was analysed in the cancer cells to further prove the apoptosis behaviour of respective formulations (Fig. 6a, b). Consistent with apoptosis analysis, PD/IFS showed a significantly (*p* < 0.01) higher expression of caspase 3 in MG63 cancer cells in a concentration dependent manner. Similar trends were observed in Saos-2 cancer cells however, caspase-3 level was relatively than expressed in MG63 cells.

## Discussion

**Fig. 7:**
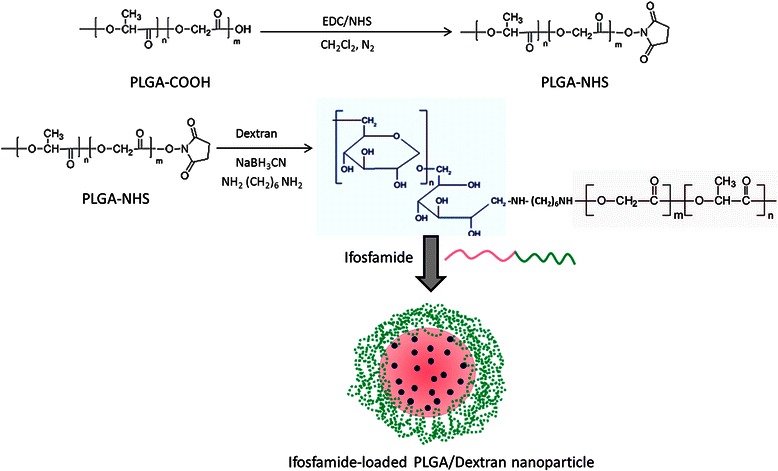
Schematic illustration of conjugation of PLGA polymer with the dextran block. The ifosfamide and block copolymer self-assembled to form the polymeric nanoparticles

Osteosarcoma (OS) is one of the typical bone cancers that occur in distal femur and proximal tibia. Although technological advancement increased the 5-year survival rate to 65 %, yet it is way behind the overall cancer survival rate. Furthermore, the metastatic or recurring disease 5-year survival rate is still at a meager 20 %. At present, the therapies for OS treatment include surgical resection followed by chemotherapy regimens of various drugs including doxorubicin, cisplatin, and ifosfamide. Specifically, IFS, a DNA-alkylating agent is indicated as a mainline treatment for OS. IFS crosslinks DNA strands and inhibits DNA replication and ultimately leads to apoptosis due to activation of caspases in the cells. In order to increase its therapeutic efficacy, it has to be loaded in nanoparticle-based delivery systems. A self-assembled polymeric nanoparticle which has a hydrophobic core, involves in the drug incorporation and the outer hydrophilic shell prevents the delivery system against reticuloendothelial system (RES). In this study, PLGA-dextran copolymer was synthesized and used to encapsulate IFS. Biodegradable polymer, poly(lactic-co-glycolic acid) (PLGA) was selected due to its excellent systemic characteristics and biodegradability. Dextran was selected due to its hydrophilic nature and biocompatibility. Dextran has an advantage, in that it has a confluent functional (hydroxyl) group in its chain, and the hydroxyl group can be used for chemical modification with targeting moieties. To conjugate PLGA copolymer, −COOH group of PLGA was activated by means of NHS to form PLGA-NHS. This PLGA-NHS was then mixed with aminated dextran to form block copolymer (Fig. 7). We expected that incorporation of IFS in PLGA-dextran based polymeric nanoparticles will effectively increase the chemotherapeutic efficacy in cancers while at the same time reduce the overall side effects.

One of the most important criteria for successful cancer targeting is the development of a biocompatible and safe nanoparticulate system. The biocompatibility of PLGA-dextran blank NP was studied in MG63 and Saos-2 osteosarcoma cancer cells. The results clearly showed that synthesized polymers were highly biocompatible and showed a cell viability of more than 90 % throughout all the concentrations tested.

Cytotoxic potential of free IFS and PD/IFS was evaluated in both the osteosarcoma cancer cell lines. Throughout all the concentrations, PD/IFS showed significant anticancer effect than comparing to free IFS. IC50 values of free IFS and PD/IFS NP were determined to quantify the cytotoxic effect. IC50 value of free IFS and PD/IFS NP were 5.24 μg/ml and 0.932 μg/ml, respectively in MG63 cancer cells, whereas, it was 5.46 μg/ml and 1.046 μg/ml, respectively in Saos-2 cancer cells. It should be mentioned that PLGA-dextran alone did not affect cell viability. Therefore, the therapeutic efficacy is only due to the drug loaded within the nanoparticles. The nanoparticle adsorbed onto the cell membrane which resulted in an increase in the intracellular drug concentration, offering a gradient that would favour drug influx into the cells [20]. Moreover, efficient uptake of NP could be a potential contributing factor in the enhanced cytotoxic effect of delivery systems [21].

Consistent with cytotoxicity assay, PD/IFS showed a significantly (*p* < 0.01) higher apoptosis of cancer cells in MG63 cancer cells. Similar trends were observed in Saos-2 cancer cells however, apoptosis rate was relatively than MG63 cells. The difference in apoptosis rate between two cell lines could be due to the biological origin and its growth rate. Consistent with cell apoptosis, PD/IFS showed a significantly (*p* < 0.01) higher expression of caspase 3/7 in MG63 cancer cells in a concentration dependent manner. Similar trends were observed in Saos-2 cancer cells however, caspase-3 level was relatively than expressed in MG63 cells. Therefore it is clear that nanoparticulate formulation of IFS remarkably increased the therapeutic performance of anticancer drug.

In the clinical setting, anticancer drugs often lead to systemic toxicity which restricts the overall dose. A limited dose however will limit the therapeutic index of given anticancer drugs. This essentially promotes the phenomenon of multi drug resistance (MDR) in cancer cells which will further complicate the drug treatment. In this regard, EUROMOS trail shows that free drug (IFS) does not improve the post-operative chemotherapy and patients responded poorly. In addition, IFS increased the number of side effects in various patients. Therefore, we believe the incorporation of IFS in a nanoparticulate system could potentially improve its therapeutic efficacy while at the same time is expected to reduce its side effects. Our study successfully demonstrated that nanoparticulate encapsulation of antitumor agent will increase the therapeutic efficacy and exhibit a greater induction of apoptosis and cell death. It seems that the nanoparticle delivery system caused increased uptake of IFS and distribution in the nucleus resulting in the enhanced cell death [22]. Our study is consistent with previously published report that nanoparticle-drug conjugates induce stronger activation of apoptosis signalling pathways comparing to that of free drug. A thorough study on experimental animal models and different cell panel would bring more value to the osteosarcoma treatment.

## Conclusion

Ifosfamide-loaded PLGA-dextran polymeric nanoparticles (PD/IFS) were successfully developed and studied its anticancer efficacy against multiple osteosarcoma cancer cells. The drug-loaded nanoparticle was characterized in terms of size distribution, morphology, zeta potential, drug loading, release profile, cytotoxicity assay, and apoptosis induction. The formulated PD/IFS showed a high drug loading capacity and displayed a pH-sensitive release pattern, with a sustained release profile of the IFS. This property is important for all the biomedical applications including cancer chemotherapy. PD/IFS nanoparticles exhibited remarkable in vitro anticancer activity comparable to that of free IFS solution in a concentration dependent manner in MG63 and Saos-2 cancer cells. PLGA-dextran by itself did not affect cell viability of cancer cells indicating its excellent biocompatibility. The formulation exhibited significantly higher PARP and caspase-3 expression in both the cancer cells. Our study successfully demonstrated that nanoparticulate encapsulation of antitumor agent will increase the therapeutic efficacy and exhibit a greater induction of apoptosis and cell death. Thus, IFS-loaded PLGA-dextran based formulations could be a potential candidate for the treatment of osteosarcoma.
